# Impact of leisure satisfaction on perceived risk of infectious disease during the COVID-19 pandemic: evidence from new worker classes

**DOI:** 10.3389/fpubh.2023.1229571

**Published:** 2024-01-08

**Authors:** Young-Jae Kim, Young-Min Ban, Seung-Woo Kang

**Affiliations:** Department of Physical Education, Chung-Ang University, Seoul, Republic of Korea

**Keywords:** COVID-19, leisure activities, leisure satisfaction, new worker classes, perceived risk of infectious disease

## Abstract

**Introduction:**

This study examines how job market changes affect individuals’ leisure choices and satisfaction, focusing on worker classes that have undergone daily life changes (e.g., economic and social activities) due to the coronavirus disease-2019 (COVID-19) pandemic.

**Methods:**

A population-based cross-sectional study design was employed. South Korean workers aged 20–59 years answered an online survey administered from September 14 to October 4, 2021. Ultimately, 764 responses were used in the analysis. The measures used in this study consisted of factors affecting infectious disease risk perception and leisure satisfaction among the six new worker classes that emerged during the pandemic, according to socio-demographic status.

**Results:**

The results showed that among male participants, social, emotional, physical, environmental, and educational factors of leisure satisfaction were more strongly affected, with higher social, physical, and interpersonal relationships being factors in the perception of infectious disease risk. Among female participants, the interpersonal relationship factor of perceived risk was significantly affected by the social, emotional, and educational factors of leisure satisfaction. Interpersonal relationships, affected leisure satisfaction among members of Classes 1–3, who experienced no change in pay. However, for the members of Class6 who experienced a decrease in pay, the economic factor negatively affected leisure satisfaction and played a pivotal role in our findings.

**Conclusion:**

This study verified the risk factors that inhibit leisure satisfaction among new worker classes that emerged during the COVID-19 pandemic. Furthermore, the psychological health of people suffering pandemic-related financial constraints was affected, as they experienced a lower quality of life owing to reduced leisure activities and satisfaction.

## 1 Introduction

During the coronavirus disease pandemic (COVID-19) in South Korea, work and leisure activities, essential elements of human life, were significantly affected due to public health and safety measures (1). To address the pandemic, strict social distancing measures were implemented, leading to the transformation of numerous leisure activities into home-based endeavors, thereby blurring the boundaries between domestic life and leisure pursuits (2–6).

Satisfaction derived from work, wages, and leisure significantly influences overall life satisfaction (7–12). Higher incomes have been associated with a more diverse range of leisure activities, contributing to increased positive life satisfaction (13). Conversely, financial stress inhibits participation in leisure activities (14). The pandemic worsened employment challenges, resulting in an increase in temporary and low-wage jobs, further widening the wage gap among different worker classes (15). These economic disparities directly impacted leisure opportunities, especially for newly classified worker classes (16).

Additionally, stringent regulations limited gatherings to four or fewer individuals until 6 p.m., exclusively for fully vaccinated individuals. Post 6 p.m., gatherings were further restricted to a maximum of two individuals. Furthermore, restaurants, cafes, and indoor sports facilities were allowed to operate until 10 p.m (17). These measures significantly restricted leisure activities, adversely impacting leisure satisfaction and overall quality of life.

Amid the economic challenges brought on by COVID-19, individuals’ perceptions of infectious diseases as a significant threat to their financial stability led to profound psychological distress (18). The emergence of new working classes during the pandemic intensified existing inequalities (19). Professionals and managerial workers with remote work options (Classes 1–3) fared better, while Class 6 workers in sectors like retail and restaurants faced severe economic hardship (16). Economic instability directly limits leisure activities due to financial constraints, underscoring the need to explore shifts in leisure pursuits across all working classes amid pandemic-induced economic fluctuations.

**Figure 1:**
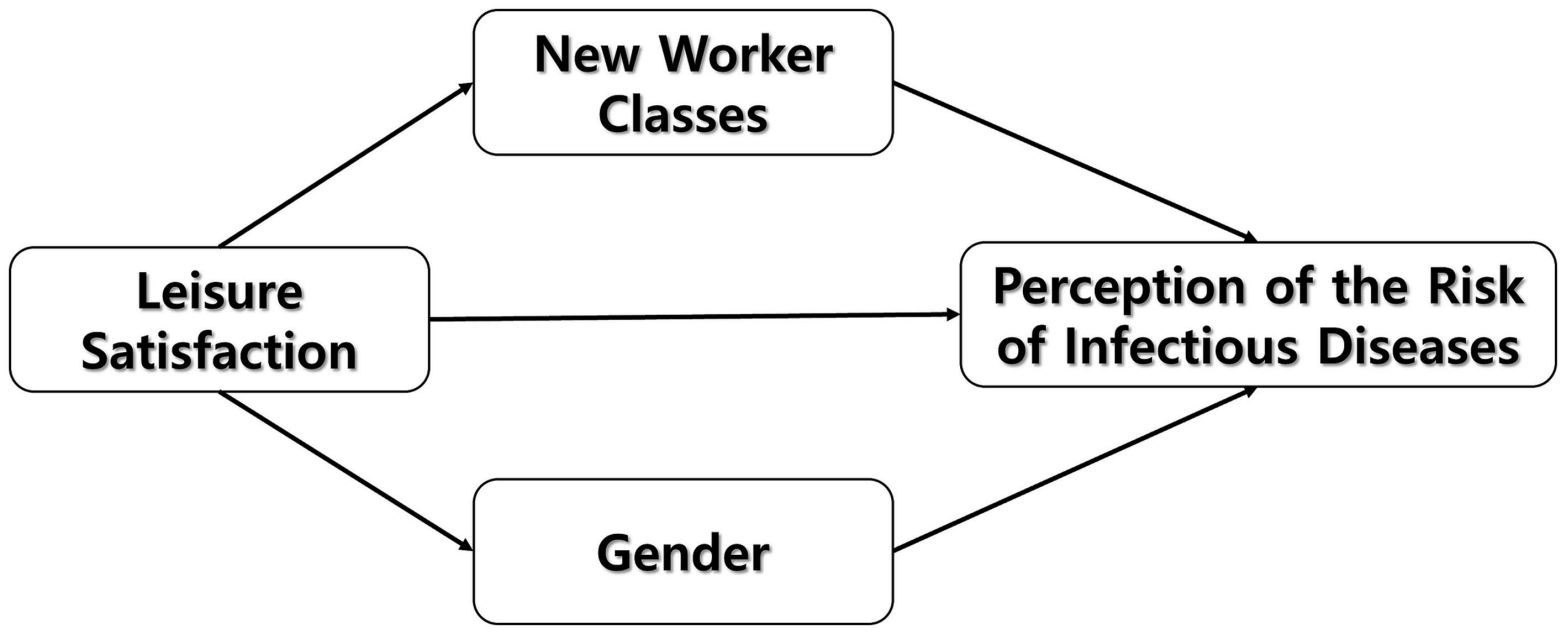
Proposed research model.

Understanding leisure needs and satisfaction is crucial for comprehending how individuals select and continue to participate in leisure activities (20–22). COVID-19 has drastically altered the landscape of leisure activities (23, 24) and satisfaction, as individuals adjusted their pursuits in response to the perceived physical and functional risks associated with the virus (20, 25–28). This study examines the transformations in leisure activities and satisfaction influenced by changes in working hours and financial conditions caused by the pandemic. Specifically, it investigates the decline in individual leisure engagement throughout the pandemic, attributing it to factors such as altered economic activities, fear of disease transmission, and adherence to social norms that discouraged physical contact. The study specifically focuses on the unique challenges faced by different working classes, aiming to discern how pandemic-induced shifts in the labor market and resulting risk perceptions influenced their leisure choices and satisfaction levels. The study findings serve as a foundation to formulate strategies to address the limitations faced by working classes in leisure activities, introduce innovative leisure pursuits, and enhance overall leisure satisfaction (Figure 1).

## 2 Methods

Workers in their 20s to 50s engaged in leisure activities were recruited through convenience sampling. The survey was administered online from September 14 to October 4, 2021, by the Korean research company Macromill Embrain. Before taking the survey, research participants were educated on the content, purpose, and ethics of the study. A survey was administered to those who agreed to participate. We gave respondents a mobile coupon worth 3,000 won (2–3 dollars) as an incentive for participating in this study. A total of 790 responses were collected, indicating a return rate of 95%. After excluding 26 surveys with multiple or missing responses, as well as those inaccurately written, 764 copies were analyzed.

### 2.1 Research participants

Reich (19) classified new classes of workers that emerged after the outbreak of COVID-19. (1) The Essentials-1: Workers maintaining their regular work hours and wages in spite of the pandemic. (2) The Essentials-2: Workers operating with reduced hours or alternating workdays while maintaining their pre-pandemic wages. (3) The Unpaid-1: Employees continuing to work as usual but receiving reduced wages. (4) The Unpaid-2: Workers operating with reduced hours or alternating workdays and receiving reduced wages. (5) The Forgotten: Employees on unpaid leave, working in temporarily closed workplaces, placed on standby, or not working at all during the pandemic. Based on this, Kim and Kang (16) classified six new classes specific to Korea and developed corresponding scales for these new classes. The demographic characteristics of the participants are presented in Table 1.

**Table 1:** Participant characteristics.

Variable			*n* (%)
Sex	Male		402 (52.6)
	Female		362 (47.4)
	Total		764
Age	20s		230 (30.1)
	30s		171 (22.4)
	40s		161 (21.1)
	50s		170 (22.3)
	60s		32 (4.2)
	Total		764
New working class	Class 1	Working from home at least 1–2 days a week with no commute or change in pay	101 (13.2)
	Class 2	Commuting to and from work without change in current pay	484 (43.4)
	Class 3	Working reduced hours and commuting every other day without change in current pay	40 (5.2)
	Class 4	Commuting normally with reduced pay	48 (6.3)
	Class 5	Working reduced hours and commuting every other day with reduced pay	31 (4.1)
	Class 6	Experiencing unpaid leave (furlough), closed business, or being on standby post-COVID-19 outbreak	60 (7.9)
	Total		764
Leisure activities	Viewing and attending cultural art events		76 (9.9)
	Viewing and attending sporting events		144 (18.8)
	Sightseeing activities		46 (6.0)
	Enjoying recreational hobby activities		87 (11.4)
	Relaxing activities		347 (45.4)
	Engaging in social and other activities		64 (8.3)
	Total		764

### 2.2 Measurement instrument

The collected data were analyzed using SPSS 26.0. After completing the first draft of the survey, one professor and two PhD-holders in the field of leisure science verified its content validity to confirm item content appropriateness. A frequency analysis was conducted to investigate the variables representing socio-demographic characteristics, and an exploratory factor analysis was conducted to examine the components of infectious disease risk perception and leisure satisfaction. To verify reliability, internal consistency was assessed using Cronbach’s *α*. Multiple linear regression analysis was conducted to analyze the relationship between the risk perception of infectious disease and leisure satisfaction according to socio-demographic factors.

### 2.3 Measurement tools

The research measures used in this study captured perceptions of the risk of infectious disease, leisure, and leisure satisfaction among each new class of worker during the pandemic. The socio-demographic variables used by Kim and Kang (16) and Sun (29), were modified and supplemented pursuant to the purposes of the present study. The survey consisted of four sets of items on sex, age, new classes of workers, and leisure activities.

#### 2.3.1 Perception of the risk of infectious diseases

A scale measuring the perceived risk of fine dust (30) that was modified pursuant to the COVID-19 pandemic and a scale measuring the perception of risks related to COVID-19 (31) were used. The adapted scale comprises 11 items across four (economic, social, physical, and interpersonal) relationship factors, measured on a 5-point Likert scale ranging from 1 (“strongly disagree”) to 5 (“strongly agree”). The results of the exploratory factor analysis conducted to determine the validity of the tool are shown in Table 2. These four factors were extracted from the 11 input items. Data were examined using Bartlett’s test of sphericity and the Kaiser-Meyer-Olkin (KMO) test to determine whether the population followed a normal distribution and whether the data were appropriate for factor analysis. This resulted in a value of 0.741, an overall cumulative variance of 68.267%, and an approximate chi-squared value of 2,323.526, *p* < 0.001. However, one item (risk perception_physical1) was removed because its factor loading was lower than the reference value (0.40). The reliability of the tool used in Kim (31) was 0.711; the reliability of the tool used in the present study was 0.748, as represented by Cronbach’s alpha.

**Table 2:** Exploratory factor analysis of the perception of the risk of infectious disease.

Items		Factor				h2
		1	2	3	4	
Risk perception_social3	COVID-19-related issues are the most important issues in society.	0.828	0.054	0.181	0.000	0.722
Risk perception_social2	Society has suffered much damage due to COVID-19.	0.792	0.060	0.109	−0.032	0.644
Risk perception_social1	The degree of risk that society feels due to COVID-19 is significant.	0.712	0.121	0.124	0.042	0.538
Risk perception_economic2	Enormous financial costs have been incurred due to COVID-19.	0.055	0.880	0.160	0.020	0.804
Risk perception_ economic1	Financial damage has been caused by COVID-19.	0.091	0.855	0.124	−0.042	0.756
Risk perception_economic3	Buying facemasks due to COVID-19 has been burdensome.	0.094	0.689	0.080	0.185	0.524
Risk perception_interpersonal relationship1	COVID-19 has negatively affected my interpersonal relationships.	0.200	0.076	0.845	0.042	0.761
Risk perception_interpersonal relationship3	COVID-19 has destroyed my interpersonal relationships.	−0.019	0.280	0.800	0.075	0.724
Risk perception_interpersonal relationship2	COVID-19 has limited my social activities.	0.379	0.059	0.695	0.040	0.633
Risk perception_physical2	COVID-19 is similar to the common cold.	0.246	0.041	0.045	0.805	0.691
Risk perception_physical3	COVID-19 is a type of pneumonia.	−0.242	0.098	0.074	0.786	0.712
Reliability		0.734	0.767	0.749	0.461	0.748
Eigenvalue		2.143	2.100	1.952	1.314	
Variate (%)		19.483	19.094	17.745	11.945	
Cumulative variance (%)		19.483	38.577	56.322	68.267	

KMO = 0.741, *χ*^2^ = 2323.526, df = 55, *p* < 0.001. KMO, Kaiser-Meyer-Olkin.

#### 2.3.2 Leisure satisfaction

Leisure satisfaction was measured using the Korean Leisure Satisfaction Scale (KLSS) (21) based on the Leisure Satisfaction Scale (LSS) (32). The original LSS consists of psychological, educational, physical, relaxational, physiological, and environmental factors, whereas the KLSS includes five factors: social, emotional, physical, environmental, and educational. This study used a survey consisting of 24 items, each measured on a 5-point Likert scale ranging from 1 (“strongly disagree”) to 5 (“strongly agree”). The results of the exploratory factor analysis conducted to verify the validity of the tool for measuring leisure satisfaction are shown in Table 3.

**Table 3:** Exploratory factor analysis of leisure satisfaction.

Items		Factor					h2
		1	2	3	4	5	
Leisure satisfaction_social3	I have made many of my current friends through leisure activities.	0.835	0.101	0.146	0.133	0.181	0.779
Leisure satisfaction_social4	During leisure activities, I cultivate friendships with people actively participating.	0.809	0.164	0.190	0.123	0.218	0.780
Leisure satisfaction_social2	I socially interact with others through leisure activities.	0.806	0.231	0.110	0.167	0.086	0.750
Leisure satisfaction_social6	I meet people who share my interests through leisure activities.	0.724	0.206	0.232	0.083	0.204	0.669
Leisure satisfaction_social5	I prefer leisure activities I can enjoy with others.	0.716	0.161	0.260	0.052	0.181	0.641
Leisure satisfaction_social1	Leisure activities help me maintain close relationships with others.	0.653	0.388	0.074	0.178	0.035	0.616
Leisure satisfaction_emotional4	I feel the vitality of life through leisure activities.	0.215	0.799	0.242	0.183	0.212	0.822
Leisure satisfaction_emotional2	Leisure activities make ordinary life less stifling.	0.270	0.799	0.173	0.219	0.151	0.789
Leisure satisfaction_emotional3	I recover psychologically through leisure activities.	0.207	0.798	−0.032	0.094	0.228	0.810
Leisure satisfaction_emotional5	I feel relaxed through leisure activities.	0.174	0.772	0.254	0.214	0.214	0.783
Leisure satisfaction_emotional1	I gain psychological stability through leisure activities.	0.272	0.753	0.175	0.152	0.162	0.721
Leisure satisfaction_physical4	I regulate my body (weight, height) through leisure activities.	0.140	0.040	0.828	0.118	0.151	0.744
Leisure satisfaction_physical3	I can test my physical strength through leisure activities.	0.221	0.156	0.810	0.164	0.196	0.795
Leisure satisfaction_physical2	The leisure activities I engage in help maintain my physical health.	0.232	0.284	0.770	0.223	0.097	0.787
Leisure satisfaction_physical1	My leisure activities can improve my physical strength.	0.203	0.290	0.753	0.241	0.160	0.776
Leisure satisfaction_physical5	My leisure activities restore my physical vitality.	0.202	0.315	0.698	0.334	0.080	0.745
Leisure satisfaction_environmental3	The facilities and places where I engage in leisure activities are fresh and clean.	0.135	0.198	0.200	0.829	0.216	0.832
Leisure satisfaction_environmental2	The facilities where I engage in leisure activities are well-decorated.	0.148	0.232	0.245	0.790	0.228	0.812
Leisure satisfaction_environmental4	The facilities and places where I engage in leisure activities drive interest.	0.195	0.215	0.202	0.759	0.284	0.781
Leisure satisfaction_environmental1	The facilities and places where I engage in leisure activities are visually appealing.	0.156	0.238	0.321	0.713	0.253	0.756
Leisure satisfaction_educational3	My leisure activities allow me to indirectly experience what I cannot experience in real life.	0.164	0.183	0.123	0.153	0.823	0.777
Leisure satisfaction_educational2	My leisure activities allow me to obtain new information.	0.196	0.256	0.145	0.242	0.756	0.754
Leisure satisfaction_educational1	My leisure activities expose me to diverse cultures.	0.184	0.191	0.149	0.348	0.729	0.781
Leisure satisfaction_educational4	My leisure activities help me learn about society in general.	0.257	0.192	0.215	0.217	0.723	0.718
Reliability		0.908	0.931	0.916	0.913	0.885	0.951
Eigenvalue		4.204	4.044	3.739	3.192	3.003	
Variate (%)		17.515	16.850	15.581	13.301	12.513	
Cumulative variance (%)		17.515	34.365	49.946	63.247	75.760	

KMO = 0.888, χ^2^ = 5949.643, df = 91, *p* < 0.001. KMO, Kaiser-Meyer-Olkin.

To determine whether the population followed a normal distribution and whether the data were appropriate for factor analysis, Bartlett’s test of sphericity and the KMO test were applied. According to Bartlett’s test, the resulting value was 0.888, overall cumulative variance was 75.760%, and approximate chi-squared value was 5,949.643, *p* < 0.001. The reliability of each factor of the tool used in Lee and Kim (30) was above 0.60, with social satisfaction at 0.908, emotional satisfaction at 0.931, physical satisfaction at 0.916, environmental satisfaction at 0.913, and educational satisfaction at 0.885. The reliability of the tool used in the present study was 0.951, as represented by the Cronbach’s alpha.

## 3 Results

This study revealed a gap in leisure activities caused by changes in economic and work conditions that formed the basis of everyday life during the COVID-19 pandemic. It also investigated how differences in infectious disease risk perception affected one’s choice of and satisfaction with leisure.

### 3.1 Relationship between leisure satisfaction and perception of infectious disease risk

The following results support the first research question, whether “infectious disease risk perception will affect leisure satisfaction,” to identify the effect of the perceived risk of infectious disease on leisure satisfaction. Table 4 shows the results of a multiple regression analysis, where the perception of the risk of infectious disease (economic, social, physical, and interpersonal relationships) was input as the independent variable, and leisure satisfaction (social, emotional, physical, environmental, and educational) was set as the dependent variable.

**Table 4:** Leisure satisfaction according to the perception of the risk of infectious disease.

Dependent variable	Independent variable: Risk perception	Unstandardized coefficient		Standardized coefficient	*t*	Collinearity statistics		*R* ^2^	*F*
		*B*	Std. Error	*β*		Tolerance	VIF		
Leisure satisfaction_social	(Constant)	2.103	0.208		10.132^***^			0.074	16.115^***^
	Economic	−0.052	0.035	−0.056	−1.487	0.137	1.154		
	Social	0.103	0.045	0.087	2.285^*^	0.847	1.181		
	Physical	0.077	0.038	0.072	2.021^*^	0.964	1.037		
	Interpersonal relationship	0.244	0.042	0.233	5.868^***^	0.773	1.294		
Leisure satisfaction_emotional	(Constant)	2.208	0.176		12.553^***^			0.125	28.274^***^
	Economic	−0.035	0.030	−0.043	−1.186	0.866	1.154		
	Social	0.246	0.038	0.237	6.446^***^	0.847	1.181		
	Physical	0.058	0.032	0.062	1.803	0.964	1.037		
	Interpersonal relationship	0.179	0.035	0.196	5.093	0.773	1.294		
Leisure satisfaction_physical	(Constant)	2.299	0.206		11.133			0.038	8.493^***^
	Economic	−0.049	0.035	−0.053	−1.399	0.866	1.154		
	Social	0.113	0.045	0.096	2.528^**^	0.847	1.181		
	Physical	0.150	0.038	0.142	3.972^***^	0.964	1.037		
	Interpersonal relationship	0.163	0.041	0.157	3.941^***^	0.773	1.294		
Leisure satisfaction_environmental	(Constant)	2.625	0.188		13.967^***^			0.074	16.115^***^
	Economic	−0.048	0.032	−0.057	−1.496	0.866	1.154		
	Social	0.130	0.041	0.122	3.173^***^	0.847	1.181		
	Physical	0.094	0.034	0.099	2.739^***^	0.964	1.037		
	Interpersonal relationship	0.094	0.038	0.101	2.504^**^	0.773	1.294		
Leisure satisfaction_educational	(Constant)	2.261	0.197		11.494^***^			0.062	13.588^***^
	Economic	−0.022	0.033	−0.025	−0.658	0.866	1.154		
	Social	0.089	0.043	0.079	2.082^*^	0.847	1.181		
	Physical	0.099	0.036	0.098	2.743^***^	0.964	1.037		
	Interpersonal relationship	0.191	0.039	0.193	4.849^***^	0.773	1.294		

***p < 0.001, **p < 0.01, *p < 0.05.

The following sub-factors of perception of infectious disease risk, predicting the social factor of leisure satisfaction, showed positive effects: perception of risk - interpersonal (*β* = 0.233, *t* = 5.868, *p* < 0.001), perception of risk - social (*β* = 0.087, *t* = 2.285, *p* < 0.05), and perception of risk - physical (*β* = 0.072, *t* = 2.021, *p* < 0.05). With the input of risk perception sub-factors to the regression equation, the explanatory power of the final regression model was 7.4% (*R*^2^ = 0.074), which was statistically significant (*F* = 16.115, *p* < 0.001). Next, perception of risk - social (*β* = 0.237, *t* = 6.446, *p* < 0.001), a sub-factor of perception of infectious disease risk that predicts the emotional factor of leisure satisfaction, was found to have a positive effect. With the input of the risk perception sub-factor to the regression equation, the explanatory power of the final regression model was 12.5% (*R*^2^ = 0.125), which was statistically significant (*F* = 28.274, *p* < 0.001).

Next, the following sub-factors of perception of infectious disease risk, predicting the physical factor in leisure satisfaction, showed positive effects: perception of risk - interpersonal (β = 0.157, *t* = 3.941, *p* < 0.001), perception of risk - physical (β = 0.142, *t* = 3.972, *p* < 0.001), and perception of risk - social (β = 0.096, *t* = 2.528, *p* < 0.01). With the input of risk perception sub-factors to the regression equation, the explanatory power of the final regression model was 3.8% (*R*^2^ = 0.038), which was statistically significant (*F* = 8.493, *p* < 0.001).

The following sub-factors of perception of infectious disease risk, predicting the environmental factor in leisure satisfaction, showed positive effects: perception of risk - social (*β* = 0.122, *t* = 3.173, *p* < 0.001), perception of risk – interpersonal (*β* = 0.101, *t* = 2.504, *p* < 0.001), and perception of risk – physical (*β* = 0.099, *t* = 2.739, *p* < 0.01). With the input of risk perception sub-factors to the regression equation, the explanatory power of the final regression model was 7.4% (*R*^2^ = 0.074), which was statistically significant (*F* = 16.115, *p* < 0.001).

The following sub-factors of perception of infectious disease risk, predicting the educational factor of leisure satisfaction, showed positive effects: perception of risk – interpersonal (*β* = 0.193, *t* = 4.849, *p* < 0.001), perception of risk – physical (*β* = 0.098, *t* = 2.743, *p* < 0.001), and perception of risk – social (*β* = 0.079, *t* = 2.082, *p* < 0.05). With the input of risk perception sub-factors to the regression equation, the explanatory power of the final regression model was 6.2% (*R*^2^ = 0.062), which was statistically significant (*F* = 13.588, *p* < 0.001).

### 3.2 Effects of the perception of infectious disease risk on leisure satisfaction according to sex

Tables 5, 6 present the results, verifying whether infectious disease perception risk, affects leisure satisfaction based on classification by sex.

**Table 5:** Effects of the perception of the risk of infectious disease on leisure satisfaction among male participants (*N* = 402).

Dependent variable	Independent variable: Perceived risk	Unstandardized coefficient		Standardized coefficient	*t*	Collinearity statistics		*R* ^2^	*F*
		*B*	Std. Error	*β*		Tolerance	VIF		
Leisure satisfaction_social	(Constant)	1.815	0.271		6.710^***^			0.125	15.278^***^
	Economic	−0.118	0.045	−0.132	−2.604^**^	0.850	1.176		
	Social	0.184	0.055	0.167	3.339^***^	0.877	1.140		
	Physical	0.100	0.049	0.097	2.044^*^	0.960	1.041		
	Interpersonal relationship	0.286	0.053	0.286	5.415^***^	0.784	1.276		
Leisure satisfaction_emotional	(Constant)	2.222	0.234		9.482^***^			0.150	18.634^***^
	Economic	−0.089	0.039	−0.113	−2.268^*^	0.850	1.176		
	Social	0.257	0.048	0.264	5.379^***^	0.877	1.140		
	Physical	0.037	0.043	0.040	0.859	0.960	1.041		
	Interpersonal relationship	0.215	0.046	0.245	4.707^***^	0.784	1.276		
Leisure satisfaction_physical	(Constant)	2.236	0.263		8.510^***^			0.085	10.293^***^
	Economic	−0.081	0.044	−0.095	−1.837	0.850	1.176		
	Social	0.155	0.054	0.148	2.899^**^	0.877	1.140		
	Physical	0.120	0.048	0.122	2.506^*^	0.960	1.041		
	Interpersonal relationship	0.206	0.051	0.217	4.014^***^	0.784	1.276		
Leisure satisfaction_environmental	(Constant)	2.317	0.250		9.257^***^			0.072	8.826^***^
	Economic	−0.074	0.042	−0.092	−1.755	0.850	1.176		
	Social	0.169	0.051	0.170	3.316^***^	0.877	1.140		
	Physical	0.105	0.045	0.113	2.311^***^	0.960	1.041		
	Interpersonal relationship	0.155	0.049	0.173	3.184^***^	0.784	1.276		
Leisure satisfaction_educational	(Constant)	1.838	0.267		6.880^***^			0.098	11.892^***^
	Economic	−0.003	0.045	−0.003	−0.065	0.850	1.176		
	Social	0.153	0.054	0.143	2.816^***^	0.877	1.140		
	Physical	0.114	0.048	0.114	2.355^*^	0.960	1.041		
	Interpersonal relationship	0.218	0.052	0.225	4.191^***^	0.784	1.276		

***p < 0.001, **p < 0.01, *p < 0.05.

**Table 6:** Effects of the perception of the risk of infectious disease on leisure satisfaction among female participants (*N* = 362).

Dependent variable	Independent variable: Perceived risk	Unstandardized coefficient		Standardized coefficient	*t*	Collinearity statistics		*R* ^2^	*F*
		*B*	Std. Error	*β*		Tolerance	VIF		
Leisure satisfaction_social	(Constant)	2.266	0.322		7.040^***^			0.043	5.023^***^
	Economic	0.030	0.054	0.030	0.551	0.880	1.137		
	Social	0.024	0.075	0.018	0.320	0.795	1.257		
	Physical	0.075	0.059	0.068	1.281	0.952	1.050		
	Interpersonal relationship	0.203	0.065	0.186	3.116^***^	0.744	1.344		
Leisure satisfaction_emotional	(Constant)	2.258	0.270		8.366^***^			0.098	10.822^***^
	Economic	0.016	0.046	0.019	0.356	0.880	1.137		
	Social	0.207	0.063	0.183	3.273^***^	0.795	1.257		
	Physical	0.086	0.049	0.090	1.761	0.952	1.050		
	Interpersonal relationship	0.152	0.055	0.162	2.789^***^	0.744	1.344		
Leisure satisfaction_physical	(Constant)	2.240	0.327		6.853^***^			0.049	5.622^***^
	Economic	0.001	0.055	0.001	0.019	0.880	1.137		
	Social	0.086	0.055	0.064	1.117	0.795	1.257		
	Physical	0.198	0.059	0.175	3.329^***^	0.952	1.050		
	Interpersonal relationship	0.108	0.066	0.097	1.624	0.744	1.344		
Leisure satisfaction_environmental	(Constant)	2.882	0.289		9.984^***^			0.011	1.973
	Economic	−0.019	0.049	−0.022	−0.389	0.880	1.137		
	Social	0.091	0.068	0.079	1.349	0.795	1.257		
	Physical	0.099	0.053	0.101	1.881	0.952	1.050		
	Interpersonal relationship	0.032	0.058	0.033	0.546	0.744	1.344		
Leisure satisfaction_educational	(Constant)	2.655	0.297		8.942^***^			0.098	11.892^***^
	Economic	−0.037	0.050	−0.041	−0.746	0.880	1.137		
	Social	0.023	0.070	0.019	0.333	0.795	1.257		
	Physical	0.097	0.054	0.095	1.796	0.952	1.050		
	Interpersonal relationship	0.163	0.060	0.163	2.716^***^	0.744	1.344		

***p < 0.001, **p < 0.01, *p < 0.05.

According to the research results, the social factor in leisure satisfaction for male participants was positively related to the following sub-factors of perceived infectious disease risk: perception of risk - interpersonal relationship (*β* = 0.286, *t* = 5.415, *p* < 0.001), perception of risk – social (*β* = 0.167, *t* = 3.339, *p* < 0.001), and perception of risk – physical (*β* = 0.097, *t* = 2.044, *p* < 0.05). With the input of risk perception sub-factors to the regression equation, the explanatory power of the final regression model was 12.5% (*R*^2^ = 0.125), which was statistically significant (*F* = 15.278, *p* < 0.001).

The emotional factor in leisure satisfaction was positively affected by perception of risk - social, a sub-factor of perception of infectious disease risk (*β* = 0.264, *t* = 5.379, *p* < 0.001). With the input of this sub-factor in the regression equation, the explanatory power of the final regression model was 15.0% (*R*^2^ = 0.150), which was statistically significant (*F* = 28.274, *p* < 0.001).

The physical factor in leisure satisfaction was positively related to the following sub-factors of perception of infectious disease risk: perception of risk – interpersonal (*β* = 0.217, *t* = 4.014, *p* < 0.001), perception of risk – social (*β* = 0.148, *t* = 2.899, *p* < 0.01), and perception of risk - physical (*β* = 0.122, *t* = 2.506, *p* < 0.05). With the input of risk perception sub-factors on the regression equation, the explanatory power of the final regression model was 8.5% (*R*^2^ = 0.085), which was statistically significant (*F* = 10.293, *p* < 0.001).

The environmental factor in leisure satisfaction was positively related to the following sub-factors of the perception of infectious disease risk: perception of risk – interpersonal (*β* = 0.173, *t* = 3.184, *p* < 0.001), perception of risk – social (*β* = 0.170, *t* = 3.316, *p* < 0.001), and perception of risk - physical (*β* = 0.113, *t* = 2.311, *p* < 0.001). With the input of risk perception sub-factors on the regression equation, the explanatory power of the final regression model was 7.2% (*R*^2^ = 0.072), which was statistically significant (*F* = 8.826, *p* < 0.001).

Finally, the educational factor in leisure satisfaction was positively related to the following sub-factors of the perception of infectious disease risk: perception of risk – interpersonal (*β* = 0.225, *t* = 4.191, *p* < 0.001), perception of risk – social (*β* = 0.143, *t* = 2.816, *p* < 0.001), and perception of risk – physical (*β* = 0.114, *t* = 2.355, *p* < 0.05). With the input of risk perception sub-factors on the regression equation, the explanatory power of the final regression model was 9.8% (*R*^2^ = 0.098), which was statistically significant (*F* = 11.893, *p* < 0.001).

Among female participants, the social factor in leisure satisfaction was positively affected by the perception of risk - interpersonal, a sub-variable of perception of infectious disease risk (*β* = 0.186, *t =* 3.116, *p* < 0.001). With the input of the risk perception sub-factor on the regression equation, the explanatory power of the final regression model was 4.3% (*R^2^* = 0.043), which was statistically significant (*F* = 5.023, *p* < 0.001).

The emotional factor in leisure satisfaction was positively related to the following sub-factors of perception of infectious disease risk: perception of risk – social (*β* = 0.183, *t* = 3.273, *p* < 0.001) and perception of risk - interpersonal (*β* = 0.162, *t* = 2.789, *p* < 0.001). With the input of risk perception sub-factors on the regression equation, the explanatory power of the final regression model was 9.8% (*R*^2^ = 0.098), which was statistically significant (*F* = 10.822, *p* < 0.001).

Next, the physical factor in leisure satisfaction was positively related to perception of risk - physical, a sub-variable of perception of infectious disease risk (*β* = 0.175, *t* = 3.329, *p* < 0.001). With the input of the risk perception sub-factor on the regression equation, the explanatory power of the final regression model was 4.9% (*R*^2^ = 0.049), which was statistically significant (*F* = 5.622, *p* < 0.001).

Finally, the educational factor in leisure satisfaction was also positively related to perception of risk – interpersonal (*β* = 0.163, *t* = 2.716, *p* < 0.001), a sub-variable of perception of infectious disease risk. With the input of the risk perception sub-factor to the regression equation, the explanatory power of the final regression model was 9.8% (*R*^2^ = 0.098), which was statistically significant (*F* = 11.892, *p* < 0.001).

### 3.3 Effects of the perception of infectious disease risk on leisure satisfaction according to new worker classes

Table 7 presents the results of verifying whether the proposition that “infectious disease risk perception, according to the characteristics of new worker classes, will affect leisure satisfaction” can examine the effects of the perception of infectious disease risk on leisure satisfaction according to socio-demographic characteristics.

**Table 7:** Effects of the perception of the risk of infectious disease by characteristics of new classes of workers on leisure satisfaction (*N* = 764).

Selected variable	Dependent variable	Independent variable perceived risk	Unstandardized coefficient		Standardized coefficient	*t*	Collinearity statistics		*R* ^2^	*F*
			*B*	Std. Error	*β*		Tolerance	VIF		
Class 1	Leisure satisfaction	(Constant)	9.515	1.972		4.824^***^			0.238	8.820^***^
		Economic	0.074	0.301	0.023	0.245	0.851	1.176		
		Social	0.470	0.454	0.099	1.034	0.828	1.208		
		Physical	0.691	0.332	0.195	2.148^**^	0.928	1.078		
		Interpersonal relationships	1.338	0.337	0.392	3.968^***^	0.779	1.284		
Class 2		(Constant)	11.935	1.050		11.370^***^			0.072	10.399^***^
		Economic	−0.281	0.176	−0.074	−1.598	0.894	1.118		
		Social	0.581	0.212	0.127	2.744^***^	0.897	1.115		
		Physical	0.575	0.192	0.133	2.987^***^	0.972	1.029		
		Interpersonal relationships	0.770	0.201	0.185	3.840^***^	0.826	1.211		
Class 3		(Constant)	10.764	2.701		3.985^***^			0.356	6.384^***^
		Economic	−0.581	0.529	−0.157	−1.098	0.841	1.189		
		Social	0.951	0.616	0.231	1.544	0.741	1.350		
		Physical	−0.598	0.572	−0.144	−1.046	0.874	1.145		
		Interpersonal relationships	2.195	0.617	0.568	3.557^***^	0.648	1.544		
Class 4		(Constant)	10.322	3.121		3.307^***^			0.090	2.168
		Economic	0.753	0.686	0.178	1.098	0.733	1.365		
		Social	0.689	0.764	0.162	0.914	0.613	1.632		
		Physical	−0.066	0.598	−0.016	−0.110	0.875	1.143		
		Interpersonal relationships	0.701	0.734	0.178	0.955	0.560	1.786		
Class 5		(Constant)	18.570	5.034		3.6898^***^			−0.027	0.806
		Economic	−0.653	1.039	−0.149	−0.629	0.612	1.634		
		Social	−0.436	1.058	−0.088	−0.412	0.757	1.321		
		Physical	0.911	0.627	0.276	1.453	0.951	1.052		
		Interpersonal relationships	0.587	0.852	0.181	0.689	0.497	2.013		
Class 6		(Constant)	13.208	1.866		7.077^***^			0.197	4.611^***^
		Economic	−0.856	0.333	−0.332	−2.573^**^	0.816	1.225		
		Social	1.380	0.481	0.399	2.869^***^	0.705	1.418		
		Physical	0.071	0.338	0.025	0.210	0.961	1.041		
		Interpersonal relationships	0.654	0.439	0.209	1.489	0.693	1.444		

***p < 0.001, **p < 0.01, *p < 0.05.

According to the research, the leisure satisfaction of participants in Class 1 among the new classes of workers was positively affected by the following sub-variables of perception of infectious disease risk: perception of risk – interpersonal (*β* = 0.392, *t* = 3.968, *p* < 0.001) and perception of risk - physical (*β* = 0.195, *t* = 2.148, *p* < 0.01). With the input of risk perception sub-factors to the regression equation, the explanatory power of the final regression model was 23.8% (*R*^2^ = 0.238), which was statistically significant (*F* = 8.820, *p* < 0.001).

The leisure satisfaction of participants in Class 2 was positively affected by the following sub-variables of perception of infectious disease risk: perception of risk - interpersonal (*β* = 0.185, *t* = 3.840, *p* < 0.001) and perception of risk – social (*β* = 0.127, *t* = 2.744, *p* < 0.001). With the input of risk perception sub-factors on the regression equation, the explanatory power of the final regression model was 7.2% (*R*^2^ = 0.072), which was statistically significant (*F* = 10.399, *p* < 0.001).

The leisure satisfaction of participants in Class 3 was positively affected by perception of risk - interpersonal (*β* = 0.568, *t* = 3.557, *p* < 0.001). With the input of the risk perception sub-factor on the regression equation, the explanatory power of the final regression model was 35.6% (*R*^2^ = 0.356), which was statistically significant (*F* = 6.384, *p* < 0.001).

The leisure satisfaction of participants in Class 6—those experiencing unpaid leave (furlough), closed businesses, and on standby for work—was positively affected by perception of risk – social (*β* = 0.399, *t* = 2.869, *p* < 0.001) and negatively affected by perception of risk – economic (*β* = −0.332, *t* = −2.573, *p* < 0.01). With the input of risk perception sub-factors to the regression equation, the explanatory power of the final regression model was 19.7% (*R*^2^ = 0.197), which was statistically significant (*F* = 4.611, *p* < 0.001).

## 4 Discussion

The results of this study show that infectious disease risk perception differs between men and women both in general and for each affected factor of leisure satisfaction. Specifically, male participants were greatly affected by social, emotional, physical, environmental, and educational factors of leisure satisfaction, with an increase in the social, physical, and interpersonal relationship aspects of the perception of infectious disease risk. Moreover, the interpersonal factor of risk perception among female participants significantly affected social, emotional, and educational leisure satisfaction factors. The emotional, physical, and social factors of leisure satisfaction were negatively affected by the higher social, physical, and interpersonal relationship factors of risk perception. Therefore, for male participants, the interactive relationship with other leisure participants (i.e., the social factor) plays an important role in leisure satisfaction. Among female participants, interpersonal relationships also play an important role, indicating that a sense of stability obtained through leisure activities is necessary for leisure satisfaction. Therefore, leisure satisfaction has implications for participation in leisure activities and differences in participation according to sex (26, 33, 34).

Gender and age differences in life satisfaction before and after the vaccination era may be influenced by a variety of factors outlined in prior research (35–38). Previous studies suggest that different societal roles, individual health concerns, and varying perceptions of vaccine efficacy and safety across demographic groups can play pivotal roles in these disparities (39–41). Earlier studies have highlighted how societal expectations and responsibilities shaped by gender, can significantly impact one’s perception of life satisfaction. Additionally, age-related health concerns and susceptibility to the virus may contribute to differences in life satisfaction post-vaccination.

The impact of demographic, health-related, and psychosocial factors on COVID-19 vaccine intentions has been evident. Factors such as older age, being male, involvement in the care of confirmed COVID-19 patients, chronic health conditions, higher self-confidence, collective responsibility, and acceptance of the influenza vaccination administration in 2019 were associated with a greater intention to receive the COVID-19 vaccine (42–47). According to Mo et al. (48), a possible link exists between COVID-19 vaccine intention and increased life satisfaction among some Healthcare Workers (HCWs) who received the vaccine, irrespective of gender.”

According to Min (49), married men may expect less leisure time than married women, as men have longer paid working hours; however, this applies only when the number of paid working hours is considered. Upon examining time constraints in terms of leisure, married women suffer from a serious lack of leisure time, as women’s overall work hours (such as those spent doing household labor), are overwhelmingly longer than those of men. Therefore, leisure activities and satisfaction decreased among married women. According to a national survey on leisure activities after the COVID-19 pandemic, the “percentages of continuous leisure activities by gender and age” were 39.5 and 31.4% for men and women, respectively (50). In addition, differences between sexes in external mobility and interpersonal communication have significantly increased since the implementation of social distancing policies, with more evident differences for women than men (51, 52). Thus, the factors of risk perception felt between the sexes vary. Therefore, leisure satisfaction should be addressed by implementing sex-specific measures to reduce risk perception.

Furthermore, the infectious disease risk perception in new worker classes was found to affect leisure. For participants in Classes 1–3, the interpersonal relationship factor in risk perception affected leisure satisfaction. In contrast, the economic factor of risk perception negatively affected leisure satisfaction for those in Class 6. Thus, those who suffered employment instability due to the pandemic did not enjoy leisure activities because of economic and safety concerns, leading to decreased leisure satisfaction.

Previous studies related to human labor activities, leisure activities, and life satisfaction have only examined the relationships among these variables because the broadest areas of human life can be largely divided into labor and leisure. Research on the effects of labor or leisure activities on life satisfaction includes studies on employment stability, leisure activities, life satisfaction (8, 9, 53, 54), determinants of life satisfaction or quality of life (55, 56), and labor and life satisfaction (11, 57–60). However, COVID-19 has significantly changed the current labor market. People affected by changes in their job or economic status (e.g., reduced income) due to COVID-19 demonstrate low investment rates, such as low equipment consumption for at-home physical activities (61). Moreover, people with lower incomes have been significantly negatively impacted in terms of physical activity and eating habits (62, 63).

Before the pandemic, leisure satisfaction was associated with positive mental and physical health outcomes. Engaging in leisure activities was linked to reduced stress, improved mood, and overall stronger life satisfaction. The perceived risk of infectious diseases was not a prominent factor in leisure decision-making for most people, except in regions where certain diseases are endemic (64).

During the COVID-19 pandemic, leisure activities have undergone significant global transformations. Due to strict social distancing measures, lockdowns, and fear of infection, the landscape of leisure activities shifted dramatically after the onset of the pandemic. Indoor activities with large crowds were restricted, leading to a surge in interest in outdoor and solitary pursuits. Leisure satisfaction became closely tied to activities that could be conducted safely, often individually, or within small and trusted groups. The perceived risk of infectious diseases became a crucial factor in people’s leisure decision-making, with safety taking precedence over personal activity preferences (65, 66).

After the COVID-19 pandemic, with the advent of the post-vaccination era in late 2020 and 2021, the situation regarding leisure activities and perceived risk of infectious diseases gradually improved. Vaccination efforts significantly reduced the risk of severe illness and death from COVID-19, leading to the relaxation of restrictions. People began to return to some semblance of pre-pandemic life and leisure activities. However, a certain level of caution persisted. Some individuals continued to prefer outdoor or socially distanced activities and vaccination status played a significant role in people’s comfort levels. For instance, fully vaccinated individuals felt more secure about engaging in leisure activities (67).

In this context, it is crucial to not only highlight the newfound freedom from COVID-19 due to vaccinations, but also emphasize the impact of government-mandated social distancing measures and the perception that COVID-19 might not be as dangerous as initially feared.

Furthermore, it is essential to recognize the newly classified socioeconomic class, Class 6, which was economically impacted during the pandemic. Exploring the leisure satisfaction of this class during the current phase of infectious disease recovery is essential. Post-2022 research is imperative to comprehensively understand whether the leisure satisfaction of Class 6 has returned to pre-pandemic levels, increased, remained stable, or declined compared with the period during COVID-19. Such an inquiry will highlight the evolving dynamics of leisure satisfaction, especially among economically affected groups, in the face of the ongoing challenges posed by infectious diseases such as COVID-19.

Moreover, given the continued economic challenges not only in Korea but also globally after the COVID-19 pandemic (68, 69), socioeconomic class represented by Class 6 will likely face even greater economic hardship. Considering that other socioeconomic classes may also experience financial difficulties, it is essential to conduct post-COVID-19 leisure satisfaction surveys for all classes. This research provides valuable insights into the evolving landscape of leisure satisfaction across various socioeconomic groups, offering crucial information for the development of targeted policies and support programs. In the workplace, employers can promote employee well-being and satisfaction by offering flexible work arrangements, such as designated leisure time or breaks, to alleviate time constraints and enhance work-life balance. Employers should be encouraged to foster a positive work environment that supports leisure activities and provides resources for employees to engage in meaningful and fulfilling leisure experiences (70–72), and the government should be involved in promoting leisure activities and addressing the identified barriers to leisure satisfaction. The government could collaborate with relevant stakeholders to develop and implement policies that support the provision of diverse and accessible leisure opportunities, and advocate for investment in infrastructure, facilities, and programs that cater to different demographic groups and promote leisure engagement. Additionally, we recommend the allocation of resources to support research, the monitoring of leisure trends, and the development of evidence-based strategies aimed at enhancing leisure satisfaction among different socioeconomic classes (72, 73).

## 5 Limitations of this research

Given the differing socio-demographic characteristics and risk perceptions among various worker groups, the focus on new worker classes potentially limits the study’s broader applicability. Moreover, reliance on a specific sample of new working classes raises concerns regarding the generalizability of the study findings. The sample selection process might introduce biases, affecting the study’s external validity by over- or underrepresenting certain working classes.

Furthermore, the study’s reliance on self-reported measures, including leisure satisfaction and the perceived risk of infectious diseases, poses a challenge. Self-reporting is susceptible to biases such as social desirability or recall bias, potentially leading to measurement errors and affecting the reliability of findings. Additionally, the cross-sectional nature of the study design raises questions about causality and directionality in the relationship between leisure satisfaction and perceived risk of infectious diseases. The study might not capture all influencing factors, potentially impacting the observed associations.

The absence of control variables, such as age, gender, educational level, or prior health conditions, limits the study’s depth. These variables could influence both leisure satisfaction and perceived risk, providing alternative explanations for the observed relationships. Finally, the study’s narrow focus, which is solely on the impact of leisure satisfaction during the COVID-19 pandemic, overlooks the potential influence of other factors on risk perceptions. Neglecting these elements may lead to an incomplete understanding of the dynamics involved in public health crises.

To address these limitations and enhance future research, essential steps are needed, such as considering diverse samples, employing longitudinal designs, integrating objective measures alongside self-reports, incorporating relevant control variables, and broadening the scope of the investigation.

## 6 Conclusion

Various studies have been conducted on the relationship between leisure activities and satisfaction according to labor intensity, hours, and wages. It has been found that the higher the job satisfaction, the more diverse the choices of leisure activities, and the higher the leisure satisfaction. However, owing to COVID-19’s impact on social and economic conditions, novel classes of workers have emerged, thus widening the disparity among classes. Therefore, this study investigated relationships in leisure satisfaction according to the risk perception experienced by new worker classes.

Our results showed differences between men and women in perceived risk, which resulted in different leisure satisfaction factors. Moreover, we found that physical and interpersonal relationships reduced leisure satisfaction in Classes 1–3. Economic and social risk perception factors were most significantly recognized among Class 6 workers.

This study identified the risk factors that decrease leisure satisfaction for each new class of workers during the COVID-19 pandemic, a time when leisure activities were more important than ever. Furthermore, those who were economically impacted by COVID-19 were also affected in terms of psychological health (e.g., a decreased quality of life due to reduced leisure activities and satisfaction). This study viewed the issue of workers suffering from unstable working conditions and employment problems as a national disaster in South Korea and highlighted the importance of preparing for future incidents. This study provides basic data for the promotion of leisure activities and improvement of satisfaction. Future studies should investigate what can be gained from leisure activities in terms of quality of life and physical and psychological health of the newly formed worker classes. Furthermore, plans must be devised to resolve institutional risks.

## Data availability statement

The original contributions presented in the study are included in the article/supplementary material, further inquiries can be directed to the corresponding author.

## Ethics statement

The studies involving humans were approved by Chung-Ang University Ethics Committee. The studies were conducted in accordance with the local legislation and institutional requirements. The participants provided their written informed consent to participate in this study.

## Author contributions

S-WK and Y-JK performed material preparation, data collection, and analysis. S-WK wrote the first draft of the manuscript. All authors commented on subsequent versions of the manuscript, read and approved the final manuscript, and contributed to the study’s conception and design.
